# The Roles of Genetic Polymorphisms and Human Immunodeficiency Virus Infection in Lipid Metabolism

**DOI:** 10.1155/2013/836790

**Published:** 2013-11-12

**Authors:** Elaine Regina Delicato de Almeida, Edna Maria Vissoci Reiche, Ana Paula Kallaur, Tamires Flauzino, Maria Angelica Ehara Watanabe

**Affiliations:** ^1^Department of Pathology, Clinical Analysis and Toxicology, Health Sciences Center, State University of Londrina, Avenida Robert Koch, 60, CEP 86038-440 Londrina, PR, Brazil; ^2^Pathological Sciences Postgraduate Program, Biological Sciences Center, State University of Londrina, Campus Universitário, CEP 86051-970 Londrina, PR, Brazil; ^3^Postgraduate Program of Health Sciences Center, State University of Londrina, Avenida Robert Koch, 60, CEP 86038-440 Londrina, PR, Brazil; ^4^Clinical Immunology, Clinical Analysis Laboratory, Health Sciences Center, State University of Londrina, Avenida Robert Koch, 60, CEP 86038-440 Londrina, PR, Brazil; ^5^Department of Pathological Sciences, Biological Sciences Center, State University of Londrina, Campus Universitário, CEP 86051-970 Londrina, PR, Brazil

## Abstract

Dyslipidemia has been frequently observed among individuals infected with human immunodeficiency virus type 1 (HIV-1), and factors related to HIV-1, the host, and antiretroviral therapy (ART) are involved in this phenomenon. This study reviews the roles of genetic polymorphisms, HIV-1 infection, and highly active antiretroviral therapy (HAART) in lipid metabolism. Lipid abnormalities can vary according to the HAART regimen, such as those with protease inhibitors (PIs). However, genetic factors may also be involved in dyslipidemia because not all patients receiving the same HAART regimen and with comparable demographic, virological, and immunological characteristics develop variations in the lipid profile. Polymorphisms in a large number of genes are involved in the synthesis of structural proteins, and enzymes related to lipid metabolism account for variations in the lipid profile of each individual. As some genetic polymorphisms may cause dyslipidemia, these allele variants should be investigated in HIV-1-infected patients to identify individuals with an increased risk of developing dyslipidemia during treatment with HAART, particularly during therapy with PIs. This knowledge may guide individualized treatment decisions and lead to the development of new therapeutic targets for the treatment of dyslipidemia in these patients.

## 1. Introduction

 Serum lipids have a multifactorial etiology that is determined by a large number of environmental and genetic factors [1]. Genetic and dietary factors influence serum cholesterol concentration, but detailed mechanisms of their interactions are not well known. An increase in dietary cholesterol intake raises serum cholesterol concentrations in some but not all subjects. 

 Human immunodeficiency virus type 1 (HIV-1) infected patients develop dyslipidemia, resulting in a highly atherogenic lipid profile with increased levels of total cholesterol, low-density lipoprotein cholesterol (LDL-C), and triglycerides (TG) and decreased levels of high-density lipoprotein cholesterol (HDL-C) [2]. The pathogenesis of dyslipidemia in HIV-1 infection is complex and involves factors related to the virus, the host, and to the antiretroviral therapy (ART). Moreover, HIV-1 infection and ART are associated with accelerated atherosclerosis and an increased number of cases of myocardial infarction [3].

Highly active antiretroviral therapy (HAART) consists of a combination of drugs that inhibit different stages of viral replication, and it is divided mechanistically into six classes [3] based on whether it targets the viral lifecycle or viral enzymes: nucleoside reverse transcriptase inhibitors (NRTIs), nonnucleoside reverse transcriptase inhibitors (NNRTIs), protease inhibitors (PIs), fusion inhibitor (enfuvirtide or T-20), entry inhibitor chemokine receptor 5 (CCR5) antagonist maraviroc, and HIV-1 integrase strand transfer inhibitor [4, 5]. 

The introduction of HAART in 1996 dramatically reduced the mortality and morbidity in HIV-1-infected patients, leading to prolonged and improved quality of life and making HIV-1 infection a manageable chronic disease [6]. HAART uses combination formulations containing at least three antiretroviral drugs that are extremely effective in reducing the plasma viral load of HIV-1 RNA to undetectable levels [4, 7, 8].

 However, it is increasingly clear that HIV-1-infected patients exhibit an increased risk of developing noninfectious consequences of HIV-1 infection over time. In the last few years, lipodystrophy (characterized by body fat redistribution), insulin resistance, central adiposity, and dyslipidemia have been reported in HIV-1-infected patients, and their relationships with antiretroviral drugs and HIV-1 infection are the subject of global debate and research [9]. Moreover, HAART can induce severe metabolic complications, such as insulin resistance, metabolic syndrome, lipodystrophy, and cardiovascular diseases. The metabolic effects of HAART and the risk of premature and accelerated atherosclerosis in HIV-1-infected patients are well recognized. These clinical conditions have significantly high prevalence in patients infected with HIV-1 that are treated with these drugs [10].

 The type and severity of lipid abnormalities vary according to the HAART regimen used. However, genetic factors may be involved in dyslipidemia because not all patients exposed to same HAART regimen and comparable demographic, virological, and immunological characteristics develop lipid profile variations [11–13].

 Many polymorphic variants of the genes that regulate lipid metabolism are present in humans, and more than 400 genes are candidate regulators of lipid exchange. Carriers of abnormal alleles exhibit a high risk for obesity and its associated complications, and therefore there is the interest in the association between dyslipidemia, adiposity, and other diseases with different genotypes. The genes involved in the leptin-melanocortin system of regulation of energy metabolism, protein carriers of lipids and cholesterol in the blood, and enzyme-splitting lipids are of particular interest [14].

 Genetic variations of enzymes, receptors, and apolipoproteins (apo), which are essential to LDL-C metabolism, are partially involved in the regulation of serum LDL-C and total cholesterol [15]. Recently, the genetic components of dyslipidemia have been intensively investigated. Variations in a large number of genes involved in the synthesis of structural proteins and enzymes associated with lipid metabolism account for variations in the lipid profile of each individual [1].

 Genetic variations that occur at a frequency of more than 1% in a study population are called genetic polymorphisms. The genetic basis for these variations can be a single nucleotide change in the DNA sequence, known as single nucleotide polymorphisms (SNPs), insertions or deletions (indels) of one or more base pairs [16], repeats of a large number of nucleotides (variable number of tandem repeats (VNTR) or minisatellite), and repeats of a small number of nucleotides (short tandem repeat (STR) or microsatellite). SNPs are the most common type of sequence variation in the human genome. The 10 to 30 million SNPs in humans represent 90% of all sequence variations [17].

 The effect of a polymorphism depends on its interactions with environmental factors that predispose patients to dyslipidemia, such as being overweight, physical inactivity, or smoking [18–20]. 

 There are several factors that can trigger the atherogenic process, including dyslipidemia, smoking, hypertension, diabetes mellitus, physical inactivity, obesity, and a history of premature atherosclerotic disease. However, dyslipidemia is a major risk factor for developing coronary artery disease (CAD) [21]. 

 Among the genetic factors associated with CAD are variations in the genetic loci responsible for the lipoprotein structure and metabolism and the low-density lipoprotein receptor (LDLR), which may contribute to the development of CAD. Some of these genetic variations are associated with increased serum levels of lipids, and therefore, they may be associated with a high risk of CAD [15, 22, 23]. There is a direct relationship between the onset of CAD and high LDL-C because these particles contribute to atherosclerotic plaques [24]. The opposite effect is observed when HDL-C is high. This circulating lipoprotein has the protective effect of reversing cholesterol transport and promotes a set of anti-inflammatory, antioxidant, and anticoagulant actions that inhibit atherosclerosis [25].

CAD is the main cause of mortality in many parts of the industrialized world [26]. In Brazil, CAD is the major cause of mortality and morbidity in women over the age of 40 or 50 years [27]. Hence, the early identification of subjects at risk of developing CAD is an important public health issue. Salazar et al. [28] showed that Brazilian women with CAD had elevated total serum cholesterol, TG, and LDL-C concentrations. These results confirm the well-known association between CAD and high lipid concentration. According to Salazar et al. [23], common DNA polymorphisms in genes associated with lipid metabolism are potentially important genetic markers of variation in the plasma lipid profile and thus susceptibility or resistance to CAD. 

Myocardial infarction, angina pectoris, and ischemic stroke resulting from atherosclerosis are the main causes of morbidity and mortality in adults in developed and developing countries [21]. A study showed that 38% of men and 42% of women in Brazil exhibit elevated serum cholesterol [29]. Lipid profile data and the study of polymorphisms in genes encoding structural proteins and enzymes regulating lipid metabolism reveal the prevalence of dyslipidemia in a population, allowing targeted intervention for the control and prevention of atherosclerotic diseases [1, 30]. 

The considerable improvement in the rates of morbidity and mortality among HIV-1-infected patients due to HAART has progressively transformed the infection into a chronic disease [6, 7, 31, 32]. Given the increased life expectancy of these patients, a systematic evaluation of their risk for early cardiovascular events is important [10].

 Considering the importance of determining the contribution of genetic polymorphisms to the multifactorial etiology of dyslipidemia, this study reviews the genetic polymorphisms associated with changes in serum lipids and assesses the role of these polymorphisms in lipid changes in patients with HIV-1.

## 2. Dyslipidemia in HIV-1-Infected Patients

Dyslipidemia is frequently observed in HIV-1-infected patients. Its pathogenesis is complex and includes factors related to the virus, the host, and the ART. Antiretroviral drugs are associated with a state of accelerated atherosclerosis and an increase in the number of cases of myocardial infarction [3]. Cardiovascular reactions are diverse, due to several factors, such as the HIV-1 infection itself, autoimmunity, immune response against other viral infections, neoplasms, prolonged immunosuppression, malnutrition, drug cardiotoxicity [33, 34], and hormonal changes [35].

### 2.1. The Role of HIV-1 Infection

HIV-1-associated dyslipidemia was recognized for years before the widespread use of PI-based HAART [36, 37]. Viremia-associated dyslipidemia is characterized by decreased plasma concentrations of total cholesterol, LDL-C, and HDL-C and elevated plasma TG [38–40]. Low HDL-C is correlated with immune activation early in the course of HIV-1 infection [41], the repercussions of which may extend beyond atherosclerosis because of the numerous functions of HDL-C, including antioxidant and anti-inflammatory activities [42–45]. HIV-1 is also associated with an increase in acute phase HDL that lacks the normal atheroprotective functions [46].

Cholesterol is critical for several steps in HIV-1 replication. HIV-1 decreases plasma HDL-C by impairing the cholesterol-dependent efflux transporter ATP-binding cassette protein A1 (ABCA1) in human macrophages, a condition that is highly atherogenic [47]. Additionally, the inflammation stimulates endothelial lipase and certain acute phase proteins, such as serum amyloid A. The plasma level of this enzyme in humans is inversely associated with HDL-C, and the acute phase proteins accelerate the removal of HDL-C by macrophages [45].

The dyslipidemia in HIV-1-infected patients resembles that observed in other chronic infections [48]. The chronic inflammatory processes are characterized by the production of proinflammatory cytokines, such as tumor necrosis factor *α* (TNF*α*) and interferon *α* (IFN*α*), resulting in the impaired clearance of TG-rich lipoproteins and insulin resistance [49]. Moreover, the nutritional state of HIV-1-infected patients, who may undergo weight loss and protein depletion, might contribute to reduced total plasma cholesterol, HDL-C, and LDL-C levels [38, 50].

**Figure 1:**
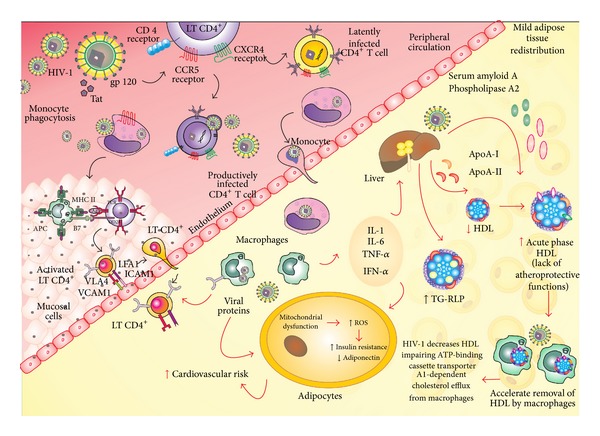
At tissues, human immunodeficiency virus type 1 (HIV-1) infects macrophages using the CD4 as receptor and the CCR5 as coreceptor and induces the local immune response. At peripheral circulation, HIV-1 infects Th1 CD4^+^ cells, particularly by the coreceptor CXCR4 that persists latently infected or becomes a productively infected cell. The viral proteins induce an proinflammatory response in peripheral circulation and in the tissues and decrease plasma high-density lipoprotein cholesterol (HDL-C) by impairing the cholesterol-dependent efflux transporter ATP-binding cassette protein A1 (ABCA1) in human macrophages, a condition that is highly atherogenic. Additionally, the viral proteins and the proinflammatory cytokines interleukin 1 (IL-1), interleukin 6 (IL-6), tumor necrosis factor *α* (TN-F*α*), and interferon *α* (IFN-*α*) stimulate endothelial lipase and certain acute phase proteins, such as serum amyloid A. The viral proteins also exert effects on the adipocytes resulting mitochondrial dysfunction, reactive oxygen species (ROS) production, and insulin resistance and decrease adiponectin. The chronic inflammatory processes increase the production of these proinflammatory cytokines, resulting in the impaired clearance of triglyceride-rich lipoproteins (TG-RLP) and insulin resistance. All these mechanisms increase the risk of cardiovascular diseases in the HIV-1-infected individuals.

Figure 1 illustrates several effects of HIV-1 infection on lipid metabolism and regulation. 

### 2.2. The Role of ART

HAART reduces the frequency of opportunistic infections and the number of AIDS-related deaths [6]. However, despite the improvements in quality of life and increased life expectancy gained with the continuous use of HAART, metabolic disorders characterized by hyperglycemia, dyslipidemia, and changes in the distribution of body fat (lipodystrophy) have been observed in HIV-1 seropositive patients [51].

The pathogenesis of HAART-related dyslipidemia is multifactorial and involves various drug-induced effects, chronic inflammatory status, hormonal influences, genetic predisposition, and HIV-1 infection itself [52]. 

The dyslipidemia associated with HAART is characterized by decreased plasma HDL-C and increased total cholesterol, TG, and LDL-C, which together constitute a highly atherogenic lipid profile [53].

HAART-related dyslipidemia appears mainly with the use of PIs. PIs may increase the hepatic synthesis of TG, VLDL-C, and to a lesser extent, cholesterol. Additionally, these drugs impair the hydrolysis of TG-rich lipoproteins by lipase, reduce free fatty acid trapping, and interfere with normal postprandial free fatty acid metabolism [54]. 

The treatment of HIV-1-infected patients is related to lipodystrophy, and dyslipidemia primarily affects those who use PIs. According to Carr et al. [55] and Chi et al. [56], over 60% of patients who are treated with PIs develop metabolic changes, such as hyperlipidemia, endothelial dysfunction, hyperglycemia, and central obesity. Persistent dyslipidemia in HIV-1-infected patients appears to be associated with increased cardiovascular risk, with a relative rate of myocardial infarction of 1.2 per year of PI exposure [57, 58]. 

One proposed mechanism of PI-induced dyslipidemia is based on the structural similarity between the catalytic region of HIV-1 protease and the LDL-receptor-related protein (LRP). This receptor is a member of the LDLR superfamily and participates in lipid metabolism. LRP normally binds to lipoprotein lipase (LPL) on the capillary endothelium, which hydrolyzes fatty acids from TG to promote free fatty acid storage in adipocytes. PIs bind to LRP due to this structural similarity and interfere with LRP-LPL complex formation; as a result, they reduce the adipose storage capacity and increase plasma TG-rich lipoproteins [59]. 

PI-induced dyslipidemia is also based upon the structural similarity with the amino acid sequence of the C-terminal region of cytoplasmic retinoic acid-binding protein type 1 (CRABP-1). During normal lipid metabolism, CRABP-1 converts retinoic acid to cis-9-retinoic acid, which binds the retinoid X receptor-peroxisome proliferator-activated receptor *γ* (RXR-PPAR*γ*) heterodimer found in adipocyte nuclei, inhibiting adipocyte apoptosis and stimulating adipocyte proliferation and differentiation. PIs likely bind to CRABP-1, increasing apoptosis and diminishing the proliferation of peripheral adipocytes [59, 60]. 

PIs also suppress the proteasome-mediated degradation of sterol regulatory element binding proteins (nSREBPs) in the liver and adipocytes. These transcription factors stimulate fatty acid and TG synthesis in the liver and adipose tissue and control several steps of cholesterol synthesis. The hepatic accumulation of nSREBPs increases TG and cholesterol biosynthesis, whereas accumulation in adipose tissue causes insulin resistance and reduced leptin expression and lipodystrophy [61].

*In vitro*, PIs and NRTIs increase the expression and secretion of proinflammatory cytokines, such as TNF-*α*, interleukin 6 (IL-6), and interleukin 1*β* (IL-1*β*), that are involved in altered adipocyte functions and decreased adiponectin. These alterations are also observed in fat and serum from HIV-1-patients with lipodystrophy that are treated with these drugs [62]. Upon entry into the cell, NRTIs are metabolized to the active triphosphorylated form and can be utilized as substrates by the mitochondrial DNA polymerase *γ*. Subsequently, they may inhibit mitochondrial DNA (mtDNA) replication and/or increase the number of mutations in mtDNA. This can lead to mtDNA depletion, the disruption of oxidative phosphorylation, decreases in ATP production, increases in reactive oxygen species, and, ultimately, inappropriate mitochondrial and cellular toxicity. 

HAART-related dyslipidemia may involve genetic predisposition, as not all patients taking HAART develop comparable metabolic disturbances [48]. In a study of 745 HIV-infected participants, Rotger et al. [30] demonstrated that 42 SNPs of genome-wide contribute to the development of dyslipidemia independent of other genetic variables, HAART, underlying conditions, sex, age, ethnicity, and HIV disease parameters. The genetic background alone explained up to 7.6% of lipid variation in HIV-infected patients (7.6% non-HDL cholesterol, 6.2% HDL-C, and 6.8% TG), and HAART alone explained up to 6.2% of lipid variation (3.9% non-HDL cholesterol, 1.5% HDL-C, and 6.2% TG). An individual with the most dyslipidemic antiretroviral and genetic background risk factors exhibits three- to fivefold increased risk of sustained dyslipidemia compared with an individual with the fewest dyslipidemic therapy and genetic background risk factors.

**Figure 2:**
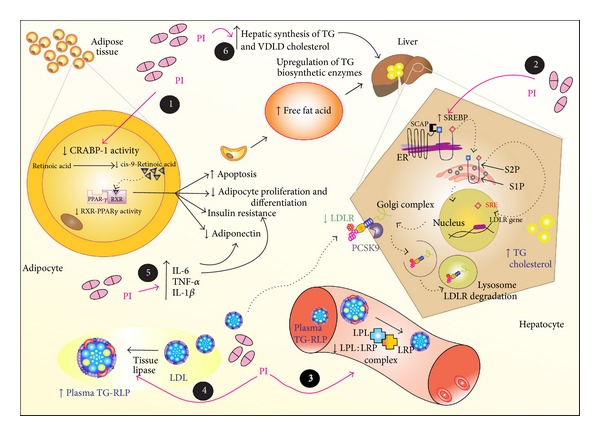
The dyslipidemia associated with protease inhibitor (PI) is characterized by decreased plasma high-density lipoprotein cholesterol (HDL-C) and increased total cholesterol, triglyceride (TG), and low-density lipoprotein cholesterol (LDL-C), which together constitute a highly atherogenic lipid profile. Several mechanisms are proposed such that: (1) the PI-induced dyslipidemia is based upon the structural similarity with the amino acid sequence of the C-terminal region of cytoplasmic retinoic acid-binding protein type 1 (CRABP-1). The PI likely binds to CRABP-1, increasing apoptosis and diminishing the proliferation of peripheral adipocytes; (2) PI suppresses the proteasome-mediated degradation of sterol regulatory element binding proteins (nSREBP) in the liver and adipocytes. These transcription factors stimulate fatty acid and TG synthesis in the liver and adipose tissue and control several steps of cholesterol synthesis. The hepatic accumulation of nSREBP increases TG and cholesterol biosynthesis, whereas accumulation in adipose tissue causes insulin resistance reduced leptin expression and lipodystrophy; (3 and 4) PI-induced dyslipidemia is also based on the structural similarity between the catalytic region of HIV-1 protease and the LDL-receptor-related protein (LRP) and interferes with LRP-LPL complex formation, as a result it reduces the adipose storage capacity and increases plasma TG-rich lipoproteins; (5) PI also increases the expression and secretion of proinflammatory cytokines, such as tumor necrosis factor alpha (TNF-*α*), interleukin 6 (IL-6), and interleukin 1*β* (IL-1*β*), which are involved in altered adipocyte functions and decreased adiponectin; and (6) PI increases the hepatic synthesis of TG, very-low density lipoprotein cholesterol (VLDL-C), and to a lesser extent, cholesterol.

**Figure 3:**
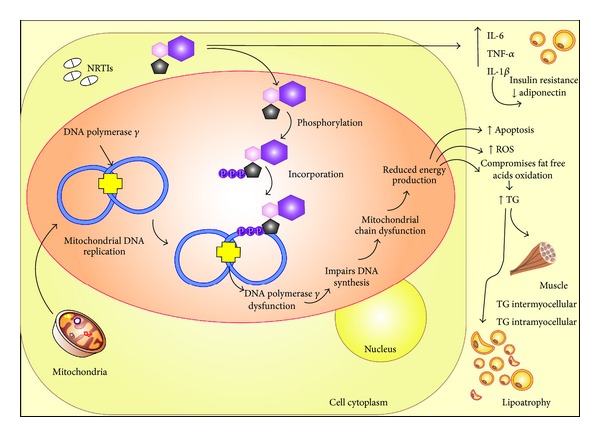
Some mechanisms are proposed to explain the effects of nucleoside reverse transcriptase inhibitors (NRTIs) in the lipid profile of human immunodeficiency virus type 1- (HIV-1-) infected individuals treated with this class of antiretroviral. (1) NRTIs increase the expression and secretion of proinflammatory cytokines, such as tumor necrosis factor alpha (TNF-*α*), interleukin 6 (IL-6), and interleukin 1*β* (IL-1*β*), that are involved in altered adipocyte function, insulin resistance, and adiponectin expression; (2) Upon entry into the cell, NRTIs are metabolized to the active triphosphorylated form and can be used as substrates by the mitochondrial DNA polymerase *γ*. Subsequently, they may inhibit mitochondrial DNA (mtDNA) replication and/or increase the number of mutations in mtDNA. This effect can lead to mtDNA depletion, the disruption of oxidative phosphorylation, decrease in ATP production, increase in reactive oxygen species (ROS), and, ultimately, inappropriate mitochondrial and cellular toxicity.

Figures 2 and 3 illustrate the main mechanisms involved in dyslipidemia associated with the PI and NRTI ART regimens, respectively. 

## 3. Genetic Polymorphisms Associated with Dyslipidemia

Polymorphisms in genes associated with dyslipidemia in patients with HIV-1 infection, either treated with ART or untreated, are reviewed. 

### 3.1. Polymorphisms in the* LDLR* Gene

The LDLR plays a major role in the removal of LDL-C particles from the blood, which, in turn, regulates cholesterol homeostasis. The LDLR modulates plasma levels of LDL-C by regulating LDL-C particle uptake by the liver. It also delivers cholesterol to the adrenal gland and gonads for steroid hormone synthesis and to the liver for bile acid synthesis [63].

Many mutations in the *LDLR* gene have been identified in patients with familial hypercholesterolemia (FH) [64–66]. Individuals with these mutations exhibit plasma cholesterol concentrations that are elevated twofold or more above normal concentrations and have an increased risk of developing atherosclerosis and CAD [63]. Considering the crucial role of LDLR in cholesterol homeostasis, SNPs in the *LDLR* gene may also contribute to the variation in plasma cholesterol levels in the general population [23].

Located on chromosome 19p13.2, the *LDLR* gene comprises 18 exons and 17 introns and encodes a protein of 839 amino acids [67]. More than 1,288 different variants in the *LDLR* gene have been reported in FH patients as follows: 55% exonic substitutions, 22% exonic small rearrangements (<100 bp), 11% large rearrangements (>100 bp), 2% promoter variants, 10% intronic variants, and 1 variant in the 3′ untranslated sequence [68].

The polymorphic nature of the *LDLR* gene has been demonstrated by its restriction fragment length polymorphisms (RFLPs) [35, 69]. The *Ava*II (T20001C, rs5925),* Hinc*II (C16730T, rs688) [23], and *Pvu*II (C>T, intron 15) polymorphisms in *LDLR *are associated with differences in serum lipid concentrations in Brazilian subjects with high risk for CAD [15].

Salazar et al. [23] investigated the effects of *LDLR* gene polymorphisms at the* Ava*II site in exon 13 (T20001C, rs5925) and the *Hinc*II site in exon 12 (C16730T, rs688) on circulating lipids of 170 unrelated white individuals presenting a lipid profile with high risk for coronary heart disease (HRG) and 130 controls. CHD subjects showed a higher frequency of the *Ava*II (A+) and* Hinc*II (H+) alleles compared with controls, and the frequency of the A+A+ (*Ava*II) and H+H+ (*Hinc*II) genotypes was greater in the HRG group than in the control group (32 versus 16% and 32 versus 18%, resp.). Moreover, in the HRG group, the A+A+ and H+H+ genotypes were associated with high concentrations of total serum cholesterol and LDL-C (*P* = 0.0001). Interestingly, neither the *Ava*II (rs5925) nor *Hinc*II (rs688) polymorphism was observed to affect serum lipid profiles in control individuals [23]. The strong association between A+A+ (*Ava*II) and H+H+ (*Hinc*II) genotypes with high total cholesterol and circulating LDL-C levels shows that *LDLR* genetic polymorphisms affect cholesterol levels in individuals with a high risk of CAD. Additionally, common polymorphisms in the *LDLR* gene are associated with inter-individual differences in plasma LDL-C levels in normal and hypercholesterolemic subjects [70–73]. 

The *Pvu*II intron 15 polymorphism is linked to other variations in *LDLR* that structurally alter the receptor activity or alter its function in a regulatory manner [73]. A *Pvu*II intron 15 polymorphism of the *LDLR *gene is associated with differences in LDL-C concentration in normal and hypercholesterolemic individuals from different countries [74, 75]. Salazar et al. [15] demonstrated the influence of *PvuII* intron 15 polymorphisms of *LDLR* on serum lipid profiles in individuals with low or high risk for CAD (HRG). The authors analyzed 128 white subjects with lipid profiles suggesting HRG and 100 white normolipidemic individuals (controls). The P1P1 genotype frequency for the *Pvu*II intron 15 polymorphism (homozygous for the absence of a restriction site) was greater in HRG-affected individuals than in control subjects (57%versus 38%, *P* < 0.05). Moreover, this genotype was strongly associated with high total cholesterol, TG, LDL-C, and VLDL-C and low HDL-C in HRG patients. Similarly, the control individuals with the P1P1 genotype presented higher concentrations of total cholesterol and LDL-C compared to those with other genotypes (P1P2 and P2P2) [15].

In a study of Brazilian Caucasian women with CAD, Salazar et al. [28] showed that the A+A+ and P1P1 homozygous genotypes (*Ava*II and *Pvu*II polymorphisms in the *LDLR* gene, resp.) were significantly higher in women with CAD than in the control group (44% versus 16%, *P* < 0.001 and 64% versus 39%, *P* < 0.05, resp.). Similarly, the frequency of the A+ and P1 alleles observed among women with CAD was higher than in controls (62% versus 44%, *P* < 0.05 and 78% versus 65%, *P* < 0.05, resp.). For the *Hinc*II polymorphism in *LDLR*, no significant difference in genotype distribution or in relative allele frequencies was observed between patients and controls.

 Salazar et al. [76] also evaluated the *Ava*II (exon 13), *Hinc*II (exon 12), and *Pvu*II intron 15 polymorphisms in 50 unrelated Brazilian individuals clinically diagnosed as FH heterozygotes and in 130 normolipidemic controls. The FH subjects showed higher frequencies of A+A+ (*Ava*II), H+H+ (*Hinc*II), and P1P1 (*Pvu*II) homozygous genotypes compared with the control group (*P* < 0.05). In addition, FH subjects presented higher frequencies of A+ (58%), H+ (61%), and P1 (78%) alleles compared with normolipidemic individuals (45%, 45%, and 64%, resp.). The strong association observed between these alleles and FH suggests that *Ava*II, *Hinc*II, and *Pvu*II polymorphisms could be useful for monitoring FH inheritance in Brazilian families.

### 3.2. *Apo E *Gene Polymorphism

The apo E protein is incorporated in the structure of HDLs-C, very low-density lipoproteins cholesterol (VLDLs-C), chylomicrons, and lipolytic degradation products (i.e., the remnants of chylomicrons and intermediate density lipoprotein cholesterol (IDL-C)). This plasma protein binds to cellular receptors. Furthermore, it is important for the transport of cholesterol and other lipids from peripheral tissues to the liver, where they are metabolized [77, 78].

Apo E is also important for the catabolism of TG-rich lipoproteins and reverse cholesterol transport in various tissues [79], which involves its binding to LDLR and the apo E hepatic receptor, the activation of enzymes including hepatic lipase, and hepatic production of VLDL-C [80, 81]. The LDLR in the liver can clear both LDL- and apo E-containing lipoproteins, but the LRP-mediated clearance of remnants is absolutely dependent on apo E [82]. Moreover, apo E influences enteral cholesterol absorption, immunoregulation, and neurobiological events such as neuronal repair, remodeling, and protection [83, 84]. 

Apo E is synthesized primarily in the liver (>90%) and also in the gut, brain, lungs, kidneys, and macrophages, and it is secreted as a glycosylated protein [83]. In addition to its important effects on lipid metabolism, vascular disease, and cholesterol modulation, apo E also regulates the growth of smooth muscle cells in the arterial wall, which impacts the progression or regression of atherosclerotic lesions [85].

The *apo E* gene is located on the long arm of chromosome 19 and encodes a protein of 299 amino acids [79]. According to Andrade and Hutz [1], the *apo E* gene exerts a strong influence on the serum levels of LDL-C.

The *apo E *gene has a common polymorphism, *Hha*I (T112C, rs429358 and C158T, rs7412), which is located in exon 4 and generates three alleles, *ε*2, *ε*3, and *ε*4; these alleles determine the six genotypes (*ε*2/*ε*2, *ε*2/*ε*3, *ε*2/*ε*4, *ε*3/*ε*3, *ε*3/*ε*4, and *ε*4/*ε*4) [79, 83]. The allele frequencies differ significantly between ethnic groups [86, 87], but *ε*3 is the most common allele in several populations [88].

According to Schwanke et al. [83], the *apo E *polymorphisms modify the protein structure and function. Apo E isoforms interact differently with lipoprotein receptors, altering their metabolism and consequently the plasma level of the circulating lipids [89].

According to Davignon et al. [90], in industrialized societies, individuals carrying the *ε*4 allele exhibit high serum levels of total cholesterol and LDL-C, while individuals carrying the *ε*3 allele exhibit intermediate levels, and those carrying the *ε*2 allele present the lowest levels. Hallman et al. [86] reported that associations between the *ε*4 allele and increased total and LDL-C levels and between the *ε*2 allele and low levels of these lipids have been documented in many studies, independently of ethnic group.

The association between *apo E* polymorphisms and CAD has been studied with regard to cardiology, as apo E affects lipoprotein metabolism and cholesterol transport [80, 81, 91]. The *apo Eε*4 allele is consistently associated with an increased risk of CAD, although its impact seems to vary according to other factors, such as gender, ethnic origin, and lifestyle [90, 92, 93]. 

Salazar et al. [28] demonstrated that the *Hha*I polymorphism in the *apo E *gene is strongly associated with CAD. Brazilian women with CAD present a higher frequency of the *ε*3/*ε*4 genotype compared with controls (40% versus 14%, *P* < 0.001). In addition, women with CAD present a higher frequency of the *ε*4 allele compared with controls (23% versus 11%, *P* < 0.05), suggesting that this allele promotes premature CAD. However, in a study of 184 Afro-Brazilian individuals, the *Hha*I polymorphism in *apo E* was not associated with hypertension or variations in serum lipid concentrations [94].

### 3.3. *Apo B* Gene Polymorphisms

 Apo B is the major protein in human LDL-C and VLDL-C, and it is synthesized in the liver and intestine. This protein is essential for the assembly, secretion, and metabolism of lipoprotein particles and for the removal of LDL-C from the circulation by LDLR on cell surfaces [63, 95]. 

Structural and genetic alterations in *apo B* are associated with defective binding to LDLR and lead to hypercholesterolemia, an important risk factor for atherosclerosis and premature CAD [96–98]. 

The *apo B *gene is located on chromosome 2p23-p24, and several mutations and SNPs are associated with either variations in plasma lipid concentrations [79] or with CAD and myocardial infarction [99–101]. The SNPs in *apo B* include the *Xba*I at exon 26 (C7673T, rs693), *Eco*RI at exon 29 (G12669A, rs1042031), *Msp*I at exon 26 (rs676210), an indel at exon 1 within the signal peptide (rs17240441), and a hypervariable region at the 3′ end (3′HVR) [102, 103]. 

Polymorphisms in the *apo B* gene, as evaluated by RFLP using the restriction enzymes *Xba*I (rs693), *Eco*RI (rs1042031), and *Msp*I (rs67210), are also associated with variability in serum cholesterol levels and coronary atherosclerosis [22, 104–106]. 

The indel, *Msp*I (rs676210),* Xba*I (rs693), and 3′HVR polymorphisms may be associated with variations in lipid levels, CAD, and myocardial infarction [104, 107–111], but these findings are controversial [112, 113].

The *Xba*I polymorphism in exon 26 of the *apo B* gene is associated with increased total cholesterol, altered postprandial lipoprotein metabolism, and increased CAD [114–117]. The *Eco*RI polymorphism in exon 29 is associated with variations in total cholesterol and TG levels, obesity, and CAD [22, 110, 118, 119]. Furthermore, the signal peptide indel polymorphism is associated with increased serum TG, total cholesterol, and LDL-C [120, 121].

Salazar et al. [28] reported that women with CAD present a higher frequency of the X-X genotype for the *Xba*I polymorphism compared with controls (42% versus 12%, *P* < 0.0001). The frequency of the X allele is also higher in women with CAD compared with controls (0.66 versus 0.39, *P* < 0.0001). The *Xba*I polymorphism is associated with increased total cholesterol, LDL-C, and CAD in Brazilian Caucasian women. 

In a study of the genotypes at three polymorphic sites of *ApoB *(the indelat the signal peptide, *Xba*I at exon 26, and *Eco*RI at exon 29), Machado et al. [122] reported the simultaneous presence of the rare *X+* and *Del* alleles (*X+*Del haplotype) in males with CHD was associated with significantly high serum levels of total cholesterol (*P* < 0.01), TG (*P* < 0.05), and LDL-cholesterol (*P* < 0.05) and with a high total cholesterol/HDL-C ratio (*P* < 0.05). These data indicate that a single haplotype, *X+Del,* within the* apo B* gene impacts lipid metabolism and may contribute to CHD susceptibility in Brazilian males.

Cavalli et al. [123] investigated four *apo B* gene polymorphisms, *Msp*I, (rs676210), *Xba*I (C7673T, rs693), the indel, and 3′HVR, in 177 white hypercholesterolemic Brazilian subjects and 100 control individuals. The genotype distribution and allele frequency of the *Msp*I, *Xba*I, and indelpolymorphisms were similar between hypercholesterolemic and control individuals, and the frequency of the alleles with ≤43 repeats in the 3′HVR was higher in the hypercholesterolemic group than in the control group (16.4 versus 8.5%, *P* < 0.05). Moreover, these alleles were associated with higher serum total cholesterol hypercholesterolemic individuals (*P* < 0.05). On the other hand, hypercholesterolemic individuals carrying at least one allele with ≤43 repeats presented higher total serum cholesterol compared with the individuals carrying both alleles with >43 repeats. In addition, an association between the indel and 3′HVR polymorphisms was observed. The alleles with ≤43 repeats and the *Del *allele were more frequent in the hypercholesterolemic individuals (*P* < 0.05). Taken together, these findings show that the *apo B* 3′HVR polymorphism may be an important genetic marker to evaluate the risk of atherosclerotic disease.

### 3.4. *Apo AI-CIII-AV* Gene Cluster Polymorphisms

Apo A-I, apo C-III, and apo A-V are mainly synthesized in the liver [124, 125]. Apo A-I is the major protein found in HDL cholesterol and is a cofactor for lecithin cholesterol acyltransferase (LCAT), the enzyme required for reverse cholesterol transport metabolism [126, 127]. The *MspI *polymorphism in the promoter region of *apo AI* is associated with differences in the plasma levels of apo AI and HDL-C [128].

ApoC-III is the major apolipoprotein of hepatic VLDL-C and; due to the role in the transport and metabolism of cholesterol, it is a candidate for determining genetic associations with serum lipid or lipoprotein levels and dyslipidemia. *In vitro* studies show that apo C-III is a noncompetitive inhibitor of LPL activity, which suggests that it plays an important role in TG-rich lipoprotein catabolism [129]. There are several polymorphisms in the *apo C-III *gene, [130]. Genetic variations in the 3′ untranslated region of *apo C-III* (*Sst*I polymorphism, rs10892152) are more frequent in hypertriglyceridemic individuals [108, 131]. 

Apo A-V is observed at lower concentrations than other apolipoproteins; however, studies have shown that it participates in TG metabolism. Apo A-V deficiency is associated with severe hypertriglyceridemia in humans because this apolipoprotein reduces plasma TG by reducing hepatic VLDL-TG production and by enhancing the lipolytic conversion of TG-rich lipoproteins [125, 132]. Three mutations in the *Apo A-V* gene have been described, at positions 148, 139, and 97 (Q148X, Q138X, and Q97X, resp.). These mutations produce three different glutamine nonsense mutations that result in Apo A-V deficiencies. 

### 3.5. *PCSK9 *Gene Polymorphisms

Another protein related to dyslipidemia is proprotein convertase subtilisin/kexin type 9 (PCSK9). The *PCSK9* gene is located on chromosome 1p32, has 12 exons, and encodes a 692 amino acid protein. There are several mutations in *PCSK9,* including c.G1120T (p.Asp374Tyr), c.T381A (p.Ser127Arg), c.T646A (p.Phe216Leu), c.A654T (p.Arg218Ser), R46L (rs11591147), and rs11206510. Mutations in *PCSK9* cause autosomal dominant hypercholesterolemia (ADH) [133]. The overexpression of PCSK9 in HepG2 cells accelerates the degradation of cell-surface LDLR through a nonproteasomal mechanism in a postendoplasmic reticulum compartment and leads to increased total cholesterol and LDL-C [134, 135]. 

### 3.6. Cholesteryl Ester Transfer Protein Gene Polymorphisms

Cholesteryl ester transfer protein (CETP) is an enzyme with a key role in HDL-C metabolism. CETP promotes the exchange of TG and cholesterol between lipoproteins, and it transfers cholesteryl esters from HDL-C to other lipoproteins for subsequent absorption of cholesterol by hepatocytes. Cholesteryl esters are transferred to LDL-Cs and VLDL-Cs in exchange for TG [136–138]. By increasing the amount of cholesteryl esters in LDL-Cs and VLDL-Cs, CETP increases the atherogenicity of these lipoproteins. High plasma CETP concentration is associated with reduced HDL-C, a strong and independent risk factor for atherosclerosis [139, 140]. 

The *CETP* gene is located on chromosome 16 and contains 16 exons [141, 142]. The protein is expressed primarily in the liver, spleen, and adipose tissue, but low levels have been detected in the small intestine, adrenal glands, heart, kidney, and skeletal muscle [143]. CETP-deficient patients exhibit elevated plasma HDL-C levels and low plasma LDL-C levels [144].

The relationship between plasma CETP, HDL-C, and atherosclerosis is complex, and *CETP* gene polymorphisms have been studied to better define this relationship [145]. Polymorphisms at the* CETP* gene locus are associated with the progression of coronary atherosclerosis independently of plasma lipase activity and HDL-C concentration.

The *Taq*IB (rs708272) polymorphism affects lipid transfer activity and HDL-C. *Taq*IB (rs708272) is one of the best studied polymorphisms in *CETP*; it consists of a silent guanine-to-adenine nucleotide substitution in intron 1. The less common allele, B2, is associated with decreased CETP activity, and in normolipemic individuals, this allele is associated with an increase in HDL-C due to decreased CETP activity [18, 146–148]. 

### 3.7. *Lipoprotein Lipase* Gene Polymorphisms

Lipoprotein lipase (LPL) is linked to the vascular endothelium and plays a crucial role in plasma lipoprotein processing. LPL catalyzes TG hydrolysis, which is the limiting step in the removal of TG-rich lipoproteins such as chylomicrons, VLDL-C, and LDL-C from the circulation [149]. LPL acts as a ligand for LDLR-related protein and for the uptake of VLDL-C and LDL-C [150].

The *LPL* gene is located on chromosome 8 (8p22), and it is composed of 10 exons [151, 152]. The known polymorphisms result in three functional variants: D9N (G28A, rs1801177), S291N (A1127G, rs268), and S447X or *Mnl*I (rs328) and two SNPs located on introns: *Hind*III at intron 8 (T381G, rs320) and *Pvu*II at intron 6 (rs285). Generally, these variants are associated with increased TG, but the S447X mutation, which truncates the last two amino acids of the polypeptide chain, decreases TG [153–155]. 

The* Hind*III (T381G, rs320) and *Pvu*II (rs285) polymorphisms, located on introns 8 and 6 of the *LPL *gene, respectively, are associated with angiographic CAD. However, Anderson et al. [156] demonstrated that *Hind*III(+) allele is moderately associated with CAD, and the *Pvu*II(−) allele is only modestly associated with CAD.

## 4. Genetic Polymorphisms Associated with Dyslipidemia in HIV-1 Infected Patients

There have been few studies of the effects of the *LDLR* gene on plasma cholesterol in HIV-1-infected patients. Tran et al. [157] showed that HIV-1 patients receiving PIs such as nelfinavir have decreased LDLR and LRP mRNA and protein levels, resulting in the reduced functional activity of these two receptors, which are involved in cholesterol metabolism. Moreover, individuals receiving nelfinavir have reduced levels of active SREBP in the nucleus.

 Plasma LDL-C levels may be influenced through the regulation of hepatic LDLR expression. The expression of LDLR is under metabolic and hormonal control. Insulin, dehydroepiandrosterone (DHEA), and growth hormone (GH) may stimulate LDLR expression and reduce plasma LDL cholesterol levels [158–160]. Petit et al. [35] evaluated the LDLR expression in HIV-patients with or without lipodystrophy. These authors found that HIV-lipodystrophy was associated with low expression of LDLR and that this decreased LDLR expression was independent of DHEA or insulin secretion.

 A study of 60 HIV-1-infected patients receiving PI therapy showed an association between *apo C-III* polymorphisms and a genetic predisposition to develop high TG and low HDL-C levels [161]; these authors suggested that *apo C-III* polymorphism genotyping could identify patients who are at risk for both hypertriglyceridemia and lipoatrophy [162]. Foulkes et al. [163] showed that there are associations between ethnic differences, *apo C-III *variants, and the development of hypertriglyceridemia in HIV-1- infected patients treated with PIs. These authors also demonstrated that Hispanics carrying the variant alleles at *apo C-III* exhibited smaller TG increases after receiving PIs compared with those carrying the wild-type genotype. According to Aragonès et al. [164], the *apo C-III* rs10892152 polymorphism predisposes HIV-1-infected patients, especially those treated with PIs, to an unfavorable lipid profile. *Apo A-V* polymorphisms also enhance PI-associated hyperlipidemia [52], and variations in this gene are risk factors for extreme hypertriglyceridemia [165].

 Tarr et al. [166] evaluated the influence of *apo C-III*, *apo E, *and *TNF *polymorphisms on the risk of ART-associated lipid disorders. No association between TNF and lipoatrophy was observed, whereas apo C-III and apo E contributed to an unfavorable lipid profile in ART-treated HIV-1 infected patients. In another study, 20 SNPs of 13 genes involved in lipid transport and metabolism were evaluated in 438 HIV-infected individuals receiving ART, and the results showed that SNPs in the *ABCA1*, *apo A-V*, and *apo C-III* genes contributed to hypertriglyceridemia, whereas SNPs in the apo* A-V *and *CETP* genes contributed to low HDL-C [11]. In a recent report by Egaña-Gorroño et al. [13], 192 SNPs in 87 genes from the lipid metabolism pathway were assessed in 727 HIV-1-infected patients starting ART. The results of this study showed that one SNP in the *apo B* gene (rs10495712) was associated with high LDL-C levels.

## 5. Conclusion

Dyslipidemia leads to atherosclerosis and CAD; thus, understanding the etiology of changes in the lipid profile is extremely important. Dyslipidemia is a complex and multifactorial condition caused by polymorphisms in genes involved in lipid metabolism and regulation and by environmental factors such as smoking, sedentary lifestyle, stress, and diet. The main genes studied in relation to dyslipidemia are those that encode proteins, receptors, and enzymes related to lipid metabolism and regulation. Polymorphisms in the *LDLR*, *apoE*, *apo B*, *apo A-I, apo C-III*, *apo A-V*, *PCSK9*, *CETP*, and *LPL* genes are associated with changes in lipid profile.

Moreover, HIV-1-infected patients often have lipid disorders. The pathogenesis of these disorders is complex and multifactorial, involving viral and host factors and ART. By itself, HIV-1 causes lipid disorders, and it acts synergistically with ART to generate dyslipidemia, insulin resistance, and lipodystrophy syndrome, especially in patients who are treated with PIs. 

The genetic causes of dyslipidemia in HIV-1-infected patients have been investigated because not all patients who use HAART exhibit metabolic disorders. Some polymorphisms in these patients are associated with lipid profile changes. Moreover, the genetic contribution to dyslipidemia alone explains up to 7.6% of the variation in HIV-1-infected patients, and HAART explains up to 6.2% of the variation. The combination of genotype and ART increases the risk of sustained dyslipidemia in HIV-1-infected individuals by up to 5-fold, with increased plasma concentrations of total cholesterol, LDL-C, and TG and decreased plasma HDL-C.

The genetic contribution to dyslipidemia is similar to or greater than the contribution of HAART. Thus, clinicians should consider genetics and the effects of ART when selecting an antiretroviral regimen for HIV-1 patients. Because gene polymorphisms cause dyslipidemia, they should be investigated in HIV-1-infected patients to identify individuals with an increased risk of developing dyslipidemia when treated with ART, especially those containing PIs. This knowledge could guide individualized treatment decisions and lead to new therapeutic targets for the treatment of dyslipidemia.
